# Mesenchymal stem cells derived from human induced pluripotent stem cells retain adequate osteogenicity and chondrogenicity but less adipogenicity

**DOI:** 10.1186/s13287-015-0137-7

**Published:** 2015-08-18

**Authors:** Ran Kang, Yan Zhou, Shuang Tan, Guangqian Zhou, Lars Aagaard, Lin Xie, Cody Bünger, Lars Bolund, Yonglun Luo

**Affiliations:** Orthopedic Research Lab, Aarhus University, 8000 Aarhus C, Denmark; Jiangsu Province Hospital on Integration of Chinese and Western Medicine, Nanjing, 210028 China; Department of Biomedicine, the Health Faculty, Aarhus University, 8000 Aarhus C, Denmark; Shenzhen Key Laboratory for Anti-aging and Regenerative Medicine, Health Science Center, Shenzhen University, 518060 Shenzhen, China

## Abstract

**Introduction:**

Previously, we established a simple method for deriving mesenchymal stem cells (MSCs) from human induced pluripotent stem cells (iPSC-MSCs). These iPSC-MSCs were capable of forming osteogenic structures in scaffolds and nanofibers. The objective of this study is to systematically characterize the mesenchymal characteristics of the iPSC-MSCs by comparing them to bone marrow-derived MSCs (BM-MSCs).

**Methods:**

Two iPSC-MSC lines (named as mRNA-iPSC-MSC-YL001 and lenti-iPSC-MSC-A001) and one BM-MSC line were used for the study. Cell proliferation, presence of mesenchymal surface markers, tri-lineage differentiation capability (osteogenesis, chondrogenesis, adipogenesis), and expression of “stemness” genes were analyzed in these MSC lines.

**Results:**

The iPSC-MSCs were similar to BM-MSCs in terms of cell morphology (fibroblast-like) and surface antigen profile: CD29+, CD44+, CD73+, CD90+, CD105+, CD11b–, CD14–, CD31–, CD34–, CD45– and HLA-DR–. A faster proliferative capability was seen in both iPSC-MSCs lines compared to the BM-MSCs. The iPSC-MSCs showed adequate capacity of osteogenesis and chondrogenesis compared to the BM-MSCs, while less adipogenic potential was found in the iPSC-MSCs. The iPSC-MSCs and the tri-lineage differentiated cells (osteoblasts, chondrocytes, adipocytes) all lack expression of “stemness” genes: *OCT4*, *SOX2*, *GDF3*, *CRIPTO*, *UTF1*, *DPPA4*, *DNMT3B*, *LIN28a*, and *SAL4*.

**Conclusions:**

The MSCs derived from human iPSCs with our method have advanced proliferation capability and adequate osteogenic and chondrogenic properties compared to BM-MSCs. However, the iPSC-MSCs were less efficient in their adipogenicity, suggesting that further modifications should be applied to our method to derive iPSC-MSCs more closely resembling the naïve BM-MSCs if necessary.

## Introduction

Mesenchymal stem cells (MSCs) have been widely used for tissue engineering due to their unique tri-lineage differentiation properties: osteogenesis, chondrogenesis, and adipogenesis [1, 2]. One attractive application of MSCs for tissue engineering is to functionalize biomaterials scaffolds by MSC co-culturing and differentiation. This can facilitate the replacement and integration of scaffolds into repairing tissues [3–5]. Currently, adult bone marrow-derived mesenchymal stem cells (BM-MSC) seem to be the most reliable cell source for tissue engineering [6]. However, limited proliferating capability of the BM-MSCs and their replicative senescence after in vitro expansion hamper their applications in a wide range of clinical applications [7].

Stem cell-derived MSCs have emerged as a significant alternative cell source to BM-MSCs. Human embryonic stem cells were mostly used for MSC derivation (ESC-MSCs), but concerns from ethical, pathological, and immunological perspectives have greatly limited the broad use of ESC-MSCs for clinical interventions. Induced pluripotent stem cells (iPSCs) generated from somatic cells represent a potentially inexhaustible cell resource with a pluripotent potential similar to ESCs. These iPSCs have the potential for patient-specific autologous therapies for diseases resulting from injury, genetic predisposition and/or aging. This approach eliminates most problems of immune incompatibility and ethical concerns. Since the first report of iPSC generation from mice and human fibroblasts by retrovirus-mediated reprogramming in 2006 and 2007 [8, 9], several methods have been established with improved safety and efficacy, such as protein transduction, mRNA reprogramming, and transgene free methods with a cocktail of small molecules [10–12].

Several attempts have been made to derive functional MSCs from iPSCs (iPSC-MSCs) [13–15]. These iPSC-MSCs obtain most mesenchymal characteristics of the naïve BM-MSCs. They are positive for typical mesenchymal markers such as CD29 (integrin beta-1), CD73 (ecto-5′-nucleotidase), CD90 (Thy-1), CD105 (endoglin), and CD146 (cell surface glycoprotein MUC18). They lack expression of CD31 (platelet endothelial cell adhesion molecule-1), CD34 (hematopoietic progenitor antigen) and CD45 (leukocyte common antigen). Most importantly, these iPSC-MSCs are capable of differentiating into three functional lineages (osteoblasts, chondrocytes and adipocytes), and diminish histoincompatibility-mediated immune responses. Moreover, the iPSC-MSCs show much greater proliferation and self-renewal capacity than BM-MSCs, which makes the iPSC-MSCs a promising substitute for BM-MSCs and ESC-MSCs. However, safety and efficacy still remain the major challenges for iPSC-MSCs.

To simplify the MSC generation procedure, we have previously established a method for iPSC-MSC derivation, which involves culturing the iPSCs directly in MSC differentiation medium followed by serial trypsinization-based passaging [16]. We have shown that the iPSC-MSCs derived by this method obtained a typical mesenchymal surface marker profile (CD73+, CD90+, CD105+, CD34–, CD45–) and were capable of osteogenesis in tissue culture vessels, synthetic three-dimensional and nanofibers. They also lack tumorigenicity when transplanted into immunodeficient mice [4, 16].

Although the iPSC-MSCs derived by our method retain the mesenchymal functions, their multipotent efficacy as compared to naïve BM-MSCs is still unknown. To further characterize the efficacy of iPSC-MSCs derived by our method, we now compare the proliferative rate, mesenchymal marker profile (CD73+, CD90+, CD105+, CD34–, CD45–), tri-lineage differentiation capacity (osteogenesis, chondrogenesis, and adipogenesis), and pluripotency of two iPSC-MSC lines and one BM-MSC line.

## Methods

### Cell culture

Normal human dermal fibroblasts (NHDFs) were kindly provided by Prof. Thomas G. Jensen from the Department of Biomedicine, Aarhus University, Aarhus, Denmark. The use of anonymous NHDFs for basic research is conducted under the guidelines from the Danish Regional Committee on Health Research Ethics. All studies were conducted in compliance with the Helsinki Declaration (http://www.wma.net/en/30publications/10policies/b3/index.html). NHDF were cultured in D10 medium consisting of Dulbecco’s modified Eagle’s medium (DMEM), 10 % fetal calf serum, 2 mM L-glutamine, and 1 % penicillin/streptomycin. All ethical approval and consent was received for the acquisition of these cells. Human iPSCs were cultured in knockout serum replacement (KSR) media, consisting of knockout DMEM (10829–018, Gibco), 20 % Knock Out Serum Replacement (10828–028, Gibco), 2 mM L-glutamine, 1 % penicillin/streptomycin, 0.1 mM nonessential amino acids (11140–050, Gibco), 0.1 mM 2-mercaptoethanol (313-50-010, Gibco) and 10 ng/ml human recombinant basic fibroblast growth factor (PHG0026, Invitrogen). The mRNA-iPSCs and mRNA-iPSC-MSC-YL001 were generated by mRNA transfection and characterized previously [16]. The human lenti-iPSCs were generated with lentivirus transduction using a single polycistronic lentiviral vector expressing OCT4, SOX2, KLF4 and c-MYC according to the methods reported by Papapetrou et al*.* [17]. Human BM-MSCs were purchased from Lonza (PT-2501) and cultured in MSC medium consisting of DMEM-low glucose (31885–023, Gibco), 10 % fetal bovine serum (FBS; 26140–079, Gibco), 2 mM L-Glutamine and 1 % penicillin/streptomycin. The K562 cells were kindly provided by Marianne Hokland from the Department of Biomedicine, Aarhus University. All cells were cultured in a tissue culture incubator with 5 % CO_2_ at 37 °C.

### Lentivirus packaging

HEK293 cells were cultured in D10 medium. At the day of transfection, 1 × 10^7^ HEK293 cells in each P15 dish (nine dishes in total) were transfected by the CaPO_4_ co-precipitation method with pRSV-REV, pMD.2G, pMDGP-Lg/pRRE plasmids and a lentiviral vector (pLM-fSV2A, Addgene ID 27512 [18]) expressing the four Yamanaka factors (OCT4, KLF4, c-Myc and SOX2) polycistronically. One day after transfection, cells were fed with fresh medium (17 ml/dish). Cell medium containing lentivirus was harvested at 48 h and 72 h post-transfection. Lentivirus was concentrated by ultra-centrifugation (25,000 rpm, 4 °C, L7 Ultracentrifuge, Beckman). Virus pellets were dissolved with phosphate-buffered saline (PBS) and stored at −80 °C. Virus titer was measured with the P24 Elisa kit (XB-1000, XpressBio).

### Lentivirus-mediated reprogramming

NHDFs (1.5 × 10^5^ cells/per well) were seeded in a six-well plate 1 day before transduction. Cells were transduced with reprogramming lentivirus in the presence of polybrene (8 μg/mL) in D10 medium. Cell media were changed every other day. Six days post-transduction, transduced NHDFs (2 × 10^4^ cells/per well) were harvested by trypsinization and seeded on irradiation-inactivated mouse feeder cells in six-well plates, and cultured in KSR medium. KSR medium was changed daily. Approximately 21 days post-transduction, the iPSC colonies were ready for picking and expansion.

### Immunofluorescence staining

For immunofluorescence staining, cells were fixed in 4 % paraformaldehyde for 20 min, followed by PBS wash (three times, 5 min each) and permeabilization with 0.3 % Triton X-100 in PBS for 10 min. The cells were then blocked with blocking solution (5 % donkey serum in PBS) at room temperature for 30 min and incubated with the primary antibodies overnight at 4 °C. Goat antihuman OCT3/4 (Abcam, ab27985, 100× diluted) and rabbit antihuman Nanog (Abcam, ab80892, 100× diluted) were used. Cells were then stained with a secondary antibody for 2 h. Alexa 594 donkey anti-goat IgG (H + L) and Alexa Fluor® 488 Donkey Anti-Rabbit IgG (H + L) (Life Technologies) were used for second antibody staining. For live cell staining of TRA-1-60 and CD44, cells were stained using the live cell imaging kit from Life Technologies (Tra-1-60 AF594, CD44 AF488) according to the manufacturer’s protocol. All images were taken with a Leica fluorescence microscope.

### Derivation of MSCs from iPSCs generated by lentiviral reprogramming

The iPSC-MSC derivation was performed according to our previous protocol. One characterized pluripotent lenti-iPSC line was used for MSC differentiation. Briefly, 3 days after passaging the lenti-iPSCs to feeder cell culture, the KSR medium was replaced with MSC medium. The lenti-iPSCs were maintained in MSC medium for 2 weeks, with medium changed every other day. Subsequently, cells were passaged to gelatin-coated (0.1 % gelatin, room temperature for 2 h) tissue culture vessels by trypsinization (0.25 % trypsin/1 mM EDTA). Cells were defined as passage 1 (P1) after the first passaging. For maintenance of iPSC-MSCs, cells were passaged when 90 % confluent and seeded with a density of 1.6 × 10^4^ cells/cm^2^ to new tissue culture vessels.

### MSC surface marker characterization by flow cytometry

Detail information on antibodies against the human antigens CD11b, CD14, CD29, CD31, CD34, CD44, CD45, CD73, CD90, CD105 and HLA-DR are shown in Table 1. Cells were harvested by trypsinization and washed with 2 % FBS-PBS twice; 2 × 10^5^ cells were re-suspended in 100 μl 2 % FBS-PBS and incubated with the conjugated antibody for 30 min at room temperature in the dark. Stained cells were then washed with 2 % FBS-PBS twice and re-suspended in 500 μl 1 % formaldehyde-PBS for flow cytometry analysis (LSRFortessa); 10,000 events were recorded for each sample and data were analyzed with Flowjo.

**Table 1:** List of flow cytometry antibodies

Antibody	Catalogue number	Dilution
CD11b FITC	eBioscience, 11–0113	40×
CD14 V450	BD, 560350	40×
CD29 APC	eBioscience, 17–0299	40×
CD31 V450	BD, 561653	40×
CD34 APC	BD, 560940	10×
CD44 Cy5.5	BD, 560531	40×
CD45 V450	BD, 560368	40×
CD73 PE	BD, 561014	10×
CD90 FITC	BD, 555595	50×
CD105 Cy5.5	BD, 560819	40×
HLA-DR Cy5.5	BD, 560652	40×

### Number of population doublings

BM-MSCs, mRNA-iPSC-MSC-YL001 and lenti-iPSC-MSC-A001 were serially subcultured in six-well plates for 2 weeks. Briefly, cells were cultured in six-well plates; when cells were 90 % confluent, they were trypsinized and counted with a hemocytometer. The cells were then cultured in a new six-well plate with the same starting cell density. The number of population doublings (NPD) was calculated according to the following equation: NPD = log10 (Nh/Ni) × 3.33, where Nh and Ni are the numbers of harvested and initiating cells, respectively [19]. NPDs were performed in six replicates for each type of MSC.

### Cell proliferation rate assay

Cell proliferation rate measurements were conducted using the Click-iT® EdU Alexa Fluor® 488 Flow Cytometry Assay Kit (Cat. no. C10425, Life Technologies). K562, BM-MSCs, mRNA-iPSC-MSC-YL001, and lenti-iPSC-MSC-A001 cells were seeded in six-well plates (1.0 × 105 cells/well) 24 h prior to changing to fresh growth medium (negative control) or growth medium containing 10 μM EdU. Cells cultured in growth medium without EdU were used as negative controls. Cells were incubated at 37 °C in a humidified incubator with 5 % CO_2_ for 2 h, then harvested by trypsinization and subjected to EdU staining according to the manufacturer’s protocol. Briefly, cells were washed with 500 μL 1 % bovine serum albumin (BSA)-PBS, incubated with 100 μL Click-iT fixative for 15 min in the dark, washed with 500 μL 1 % BSA-PBS, re-suspended with 100 μL 1× component E, stained in labeling cocktail for 30 min, washed with 500 μL 1× component E, and analyzed by flow cytometry (LSRFortessa); 10,000 events were recorded for each sample and data were analyzed with Flowjo.

### Tri-lineage differentiation

BM-MSCs, mRNA-iPSC-MSC-YL001, and lenti-iPS-MSC-A001 cells (all P9) were used for the tri-lineage differentiation (osteogenesis, chondrogenesis and adipogenesis).

For osteogenic differentiation, cells were seeded at a density of 1.2 × 10^4^ cells/cm^2^ in 96-well tissue culture plates. After 3 days, the media were changed to either MSC growth medium (as control) or osteogenic differentiation medium, which contains DMEM-high glucose (Invitrogen), 10 % FBS (Gibco), 1 % penicillin/streptomycin, 100 nM dexamethasone (D2915, Sigma), 10 mM β-Glycerophosphate (G9891, Sigma), 50 μM ascorbic acid-2 phosphate (A8960, Sigma), and 10 μM Vit-D (D1530, Sigma). Cells were maintained in growth medium or osteogenic differentiation medium for 3 weeks with medium changed every 3 days. At the end of differentiation, samples were subjected to Alizarin Red S (Sigma) staining, calcium quantification analysis following the manufacturer’s instructions, and quantitative PCR analysis.

For chondrogenic differentiation, 2.5 × 10^5^ cells were centrifuged at 500 g for 10 min to form a small pellet in a “V” shape 96-well plate. The cell pellets were cultured in either MSC growth medium (as control) or chondrogenic differentiation medium consisting of DMEM (F12, Gibco) supplemented with 10 % FBS, 50 μg/mL ascorbic acid, 100 nM dexamethasone, 1:100 ITS premix (BD Biosciences), 40 μg/ml L-proline (Sigma-Aldrich), 10 ng/mL human recombinant TGF-β3 (R&D systems), and 1 % penicillin/streptomycin [16, 20]. The medium was changed every 3 days. After 3 weeks, cell pellets were collected for toluidine blue staining to validate the extracellular chondrocyte matrix [21]. Purple stain of proteoglycans was observed by phase-contrast microscopy.

Adipogenic differentiation was carried out by culturing the cells at a density of 1.2 × 10^4^ cells/cm^2^ in DMEM-high glucose (Invitrogen) supplemented with 15 % horse serum (Sigma), and 100 nM dexamethasone (Sigma), 1 % penicillin/streptomycin (MSC growth medium as control). After 3 weeks, cells were stained with Oil-red O (Sigma-Aldrich) solution to evaluate differentiation using standard techniques as previously described [22].

### PCR and quantitative PCR

Total RNA was extracted with an RNeasy Plus Mini Kit (Qiagen) according to the manufacturer’s instructions, followed by quantification with a Nanodrop spectrophotometer and qualification by 1 % agarose gel electrophoresis. First-strand cDNA was synthesized with approximately 50 ng of total RNA using a cDNA synthesis kit (170–8891, Bio-Rad). PCR primers for stemness genes and osteogenic genes are listed in Table 2.

**Table 2:** List of primers

Gene	Primer sequences (5′–3′)	Annealing temperature (°C)	Amplicon (bp)
OCT4-F	CCTCACTTCACTGCACTGTA	58	163
OCT4-R	CAGGTTTTCTTTCCCTAGCT		
KLF4-F	GATGAACTGACCAGGCACTA	60	144
KLF4-R	GTGGGTCATATCCACTGTCT		
C-MYC-F	TGCCTCAAATTGGACTTTGG	58	191
C-MYC-R	GATTGAAATTCTGTGTAACTGC		
SOX2-F	CCCAGCAGACTTCACATGT	58	150
SOX2-R	CCTCCCATTTCCCTCGTTTT		
GDF3-F	AAATGTTTGTGTTGCGGTCA	53	178
GDF3-R	TCTGGCACAGGTGTCTTCAG		
CRIPTO-F	CGGAACTGTGAGCACGATGT	58	65
CRIPTO-R	GGGCAGCCAGGTGTCATG		
UTF1-F	CCGTCGCTGAACACCGCCCTGCTG	58	179
UTF1-R	CGCGCTGCCCAGAATGAAGCCCAC		
DPPA4-F	GGAGCCGCCTGCCCTGGAAAATTC	53	407
DPPA4-R	TTTTTCCTGATATTCTATTCCCAT		
DNMT3B-F	TGCTGCTCACAGGGCCCGATACTTC	58	170
DNMT3B-R	TCCTTTCGAGCTCAGTGCACCACAAAAC		
LIN28-F	AGTAAGCTGCACATGGAAGG	58	420
LIN28-R	ATTGTGGCTCAATTCTGTGC		
SALL4-F	GCCGTGAAGACCAATGAGAT	58	243
SALL4-R	CTCCTTCCACGCAAGTTCTC		
RUNX2-F	CAGTAGATGGACCTCGGGAA	60	188
RUNX2-R	CCTAAATCACTGAGGCGGTC		
ALP-F	ATCAGGGACATTGACGTGATC	60	137
ALP-R	TTCCAGGTGTCAACGAGGTC		
COL1A1-F	AGGGCCAAGACGAAGACATC	60	138
COL1A1-R	AGATCACGTCATCGCACAAC		
OC-F	AGTCCAGCAAAGGTGCAGCC	60	169
OC-R	TCAGCCAACTCGTCACAGTC		
GAPDH-F	TGGTATCGTGGAAGGACTCATGAC	53	189
GAPDH -R	ATGCCAGTGAGCTTCCCGTTCAGC		

For PCR characterization of the expression of stemness genes (*OCT4*, *KLF4*, *C*-*MYC*, *SOX2*, *GDF3*, *CRIPTO*, *UTF1*, *DPPA4*, *DNMT3B*, *LIN28A* and *SALL4*), PCR was performed with pfx DNA Polymerase (11708–039, Invitrogen) using 2 μl (five times diluted) cDNA as DNA temperate. The following PCR program was optimized for each stemness gene: 1 cycle of 94 °C for 3 min; 30 cycles of 94 °C for 30 seconds, 60 °C or 58 °C or 53 °C (see Table 2) for 30 seconds, and 68 °C for 30 seconds; 1 cycle at 68 °C for 7 min. The GAPDH gene was used as an endogenous control.

For quantitative PCR of osteogenic genes (of runt-related transcription factor 2 (*RUNX2*), alkaline phosphatase (*ALP*), collagen type I alpha 1 (*COL1A1*), and osteocalcin (*OC*)), 2 uL (five times diluted) cDNA was used. Quantitative PCR was performed using the LightCycle 480 SYBR Green I Master kit on LightCycler 480 (Roche) with the following PCR program: 1 hold at 95 °C for 5 min; 50 cycles at 95 °C for 10 seconds, 60 °C for 10 seconds, and 72 °C for 15 seconds. To mask the signal from primer dimers, fluorescent signal was measured at 77 °C for *RUNX2*, 80 °C for *ALP*, 82 °C for *GAPDH* and *COL1A1*, and 84 °C for *OC*. Relative gene expression was calculated using the 2^-ΔΔct^ method after normalization to the reference gene GAPDH.

### Statistics

All values are reported as mean ± SD. Data were checked for normal distribution using QQ-plots. The osteogenic gene expression between growth and osteogenic media were compared by *t*-test in each cell line. Quantitative calcium deposition was investigated using two-way analysis of variance (media type × cell line). If interactions were observed, the variables were investigated separately by the Scheffe method. *P* < 0.05 was considered statistically significant.

## Results

### MSCs from iPSCs induced by lentiviral-mediated reprogramming have the same fibroblast-like morphology as BM-MSCs

**Fig. 1:**
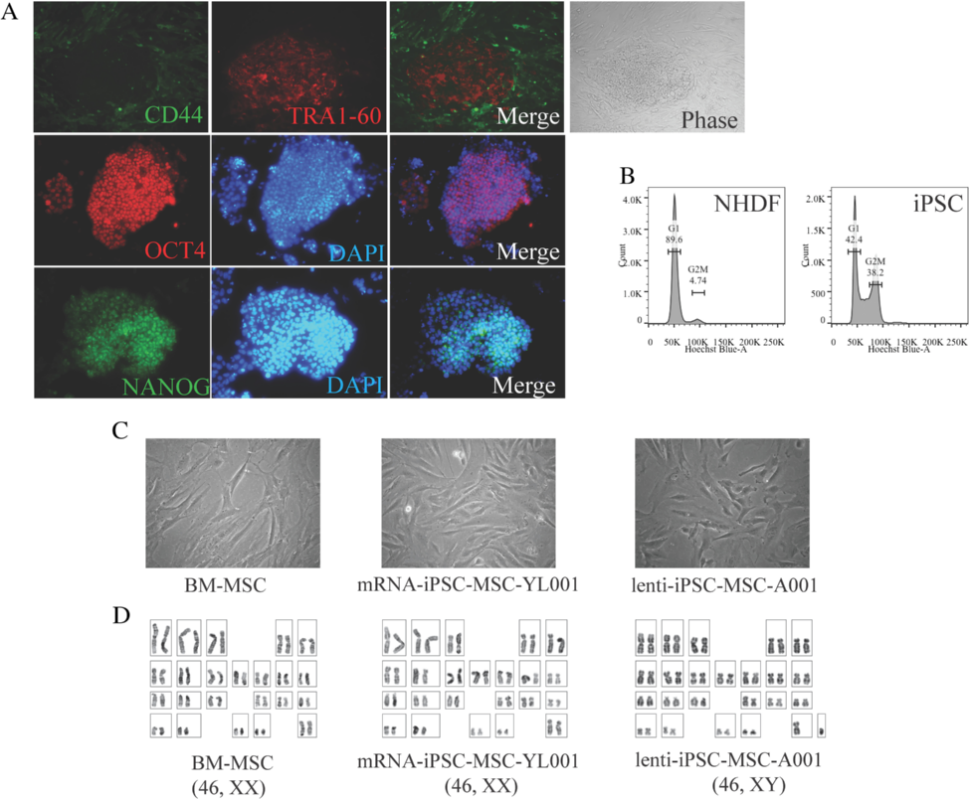
Derivation of iPSC-MSCs from iPSCs produced by lentiviral-mediated programming of normal human dermal fibroblasts. **a** One lenti-iPSC line characterized by live cell imaging of TRA-1-60+ and CD44–; immunofluorescence staining of NANOG+ and OCT3/4+, and phase-contrast image of the iPSC clone used for MSC differentiation. **b** Cell cycle analysis of the lenti-iPSC clone. **c** Phase-contrast morphology of the BM-MSC, mRNA-iPSC-MSC-YL001 and the lenti-iPSC-MSC-A001 derived from the lenti-iPSCs. Magnification 200×. **d** A representative DAPI banded karyotype result for BM-MSC (P12), mRNA-iPSC-MSC-YL001 (P13) and the lenti-iPSC-MSC-A001 (P10). *BM* bone marrow, *iPSC* induced pluripotent stem cell, *MSC* mesenchymal stem cell, *NHDF* normal human dermal fibroblast

The procedure for iPSC-MSC derivation, which we had established previously, was based on an iPSC line established by mRNA reprogramming from human fibroblasts [16]. It is unknown whether this procedure could be applied to derive functional MSCs from iPSC lines generated by other methods. Lentiviral-mediated reprogramming is favored for its high reprogramming efficiency relative to other methods [17]. We firstly generated one iPSC line using lentiviral-mediated reprogramming. The iPSC line exhibited typical stem cell morphology; expressed pluripotent cell cells markers: TRA1-60 (podocalyxin), OCT4 (octamer-binding transcription factor 4, also known as POU domain, class 5, transcription factor 1), and NANOG (Nanog homeobox) (Fig. 1a); lacked expression of CD44 (the absence of which is crucial for iPSC pluripotency (Fig. 1a)) [23]; and retained normal polyploidy (diploid as fibroblasts, Fig. 1b). We differentiated the iPSC colony into MSCs using our preciously established procedure [16]. The MSCs derived from lentivial reprogramming iPSCs (lenti-iPSC-MSCs-A001) had the same morphology (fibroblast-like) as the BM-MSCs and the mRNA-iPSC-MSC-YL001 line reported previously [16] (Fig. 1c), and are karyotypically normal (Fig. 1d).

### The iPSC-MSCs obtained similar mesenchymal surface marker profiles to BM-MSCs through in vitro cultivation

**Fig. 2:**
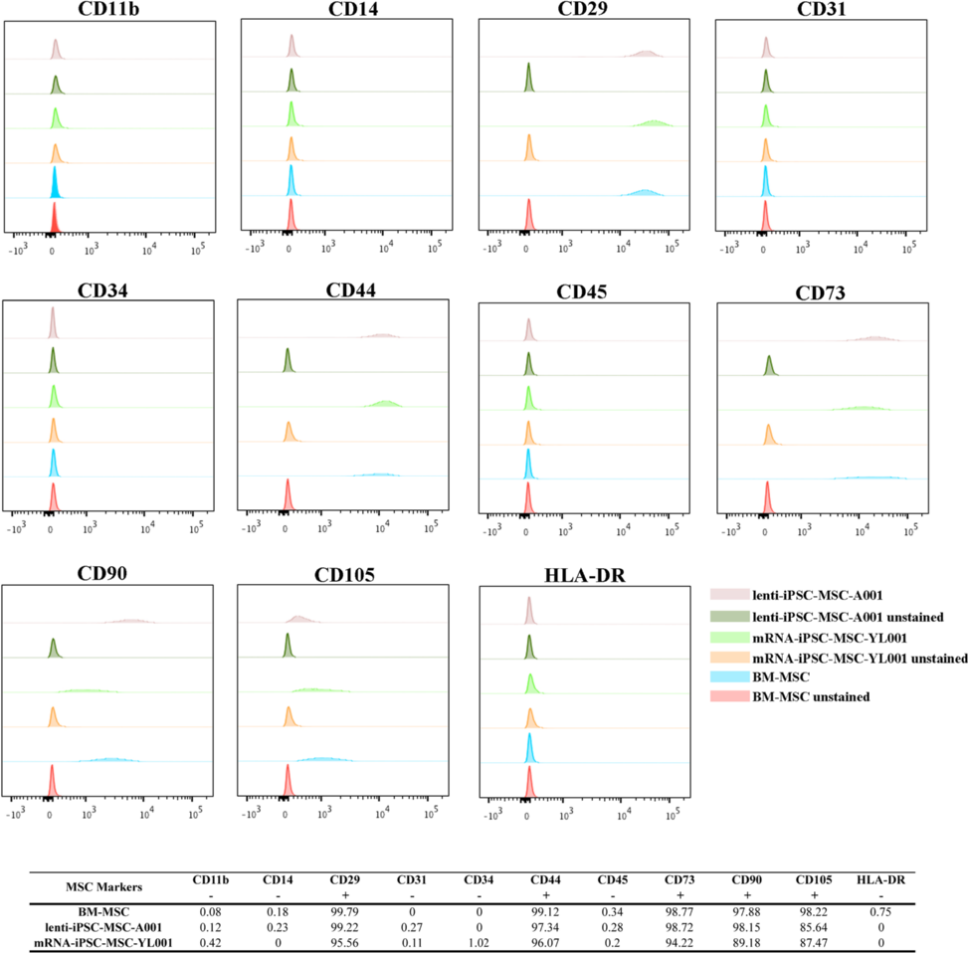
Flow cytometric analysis of mesenchymal markers CD11b–, CD14–, CD29+, CD31–, CD34–, CD44+, CD45–, CD73+, CD90+, CD105+, and HLA-DR– in BM-MSCs (P13), mRNA-iPSC-MSC-YL001 (P14), lenti-iPSC-MSC-A001 (P10). Unstained cells were used as flow cytometry control; 10,000 events were recorded for each sample. Bottom panel: percentage of cells positive for each marker is calculated by normalizing to the unstained cells. A minus value was replaced with “0”. *BM* bone marrow, *iPSC* induced pluripotent stem cell, *MSC* mesenchymal stem cell

To verify whether the iPSC-MSCs harbor mesenchymal characteristics, we analyzed the expression of five MSC-positive surface markers—CD29 (Integrin beta-1), CD44 (Phagocytic glycoprotein 1), CD73 (ecto-5′-nucleotidase), CD90 (Thy-1), and CD105 (endoglin)—and six MSC-negative surface markers—CD11b (integrin alpha-M), CD14 (monocyte differentiation antigen), CD31 (platelet endothelial cell adhesion molecule), CD34 (hematopoietic progenitor cell antigen), CD45 (T-cell surface glycoprotein), and HAL-DR (major histocompatibility complex class II antigen)—in lenti-iPSC-MSC-A001 (P10), mRNA-iPSC-MSC-YL001 (P14) and the BM-MSCs (P13) by flow cytometry. As shown in Fig. 2, the mRNA-iPSC-MSC-YL001, lenti-iPSC-MSC-A001 and BM-MSCs lacked expression of CD11b, CD14, CD31, CD34, CD45 and HLA-DR. Almost 100 % of the lenti-iPSC-MSC-A001 and mRNA-iPSC-MSC-YL001 cells were positive for CD29, CD44, and CD73, similar to that for BM-MSCs. Robust expression of CD90 and CD105 (almost 100 % positivity) was found in BM-MSCs. The abundance of CD90- and CD105-positive cells in the lenti-iPSC-MSC-A001 cells was increased following in vitro cultivation. At P3, about 70 % and 80 % of the cells were positive for CD90 and CD105, respectively (data not shown). At P13, the lenti-iPSC-MSC-A001 cells were approximately 98.15 % CD90 positive and 85.64 % CD105 positive (Fig. 2). Among the mRNA-iPSC-MSC-YL001 cells, robust CD90 and CD105 expression (89.18 % and 87.47 % positivity at P14, respectively) were observed, which is consistent with our previous observation [16]. Thus, it appears that lenti-iPSC-MSC-A001 (late passage) and mRNA-iPSC-MSC-YL001 cells display similar mesenchymal surface marker profiles as BM-MSCs. However, the MSC surface marker characterization also indicates the heterogeneity of iPSC-MSCs derived by our method, with approximately 10 % cells negative for CD105 (Fig. 2). Based on the finding of passage-dependent mesenchymal enrichment in the lenti-iPSC-MSC-A001, cells with over 6 passages were used for the following functional characterizations.

### The iPSC-MSCs possess fast proliferative capability

**Fig. 3:**
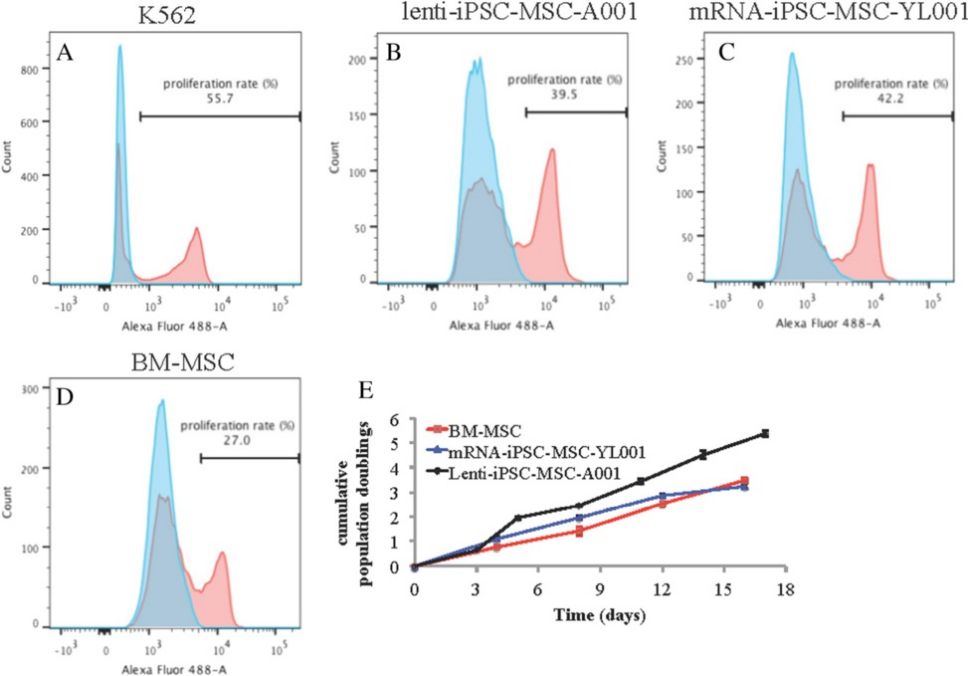
Characterization of cell proliferation and growth. Analysis of the cell proliferation rate with a Click IT cell proliferation assay in **a** control cell line (K562, chronic myeloid leukemia-derived cell line), **b** lenti-iPSC-MSC-YL001 (P7), **c** mRNA-iPSC-MSC-A001 (P7) and **d** BM-MSCs (P8). Blue plots represents negative control without EdU incubation. Pink plots represents cells treated with EdU. **e** Cumulative population doubling assay of the BM-MSCs (P11), miPSC-MSC-YL001 (P11) and lenti-iPSC-MSC-A001 (P8) for a period of 2 weeks. *BM* bone marrow, *iPSC* induced pluripotent stem cell, *MSC* mesenchymal stem cell

The iPSC-MSCs have become a promising substitute for BM-MSCs or MSCs derived from other adult tissues. The advantage of using iPSC-MSCs is attributed to their higher self-renewal capability. We compared the proliferation rate of the two iPSC-MSC lines to BM-MSCs using the Click IT-based proliferation assay and flow cytometry. K562 cancer cells were used a positive control for the assay (55.7 % proliferation rate, Fig. 3a). Higher proliferation rate was observed in the mRNA-iPSC-MSC-YL001 (42.2 %, Fig. 3b) and lenti-iPSC-MSC-A001 (39.5 %, Fig. 3c) than the BM-MSCs (27 %, Fig. 3d). The fast proliferation capacity of iPSC-MSCs was further validated by population doubling array (Fig. 3e).

### iPSC-MSCs possess adequate osteogenic and chondrogenic, but less adipogenic capabilities, compared with BM-MSCs

According to the International Society for Cellular Therapy, the capability of tri-lineage differentiation in vitro is one of the major criteria for defining MSCs [24]. We have previously shown that the mRNA-iPSC-MSC-YL001 line is capable of tri-lineage differentiation, with a focus on osteogenesis on synthetic biocompatible scaffolds [16]. However, the efficacy of tri-lineage differentiation as compared to BM-MSCs is yet to be addressed. Thus, we compared the osteogenic, chondrogenic and adipogenic differentiation capabilities of the two iPSC-MSC lines relative to the BM-MSC line.

**Fig. 4:**
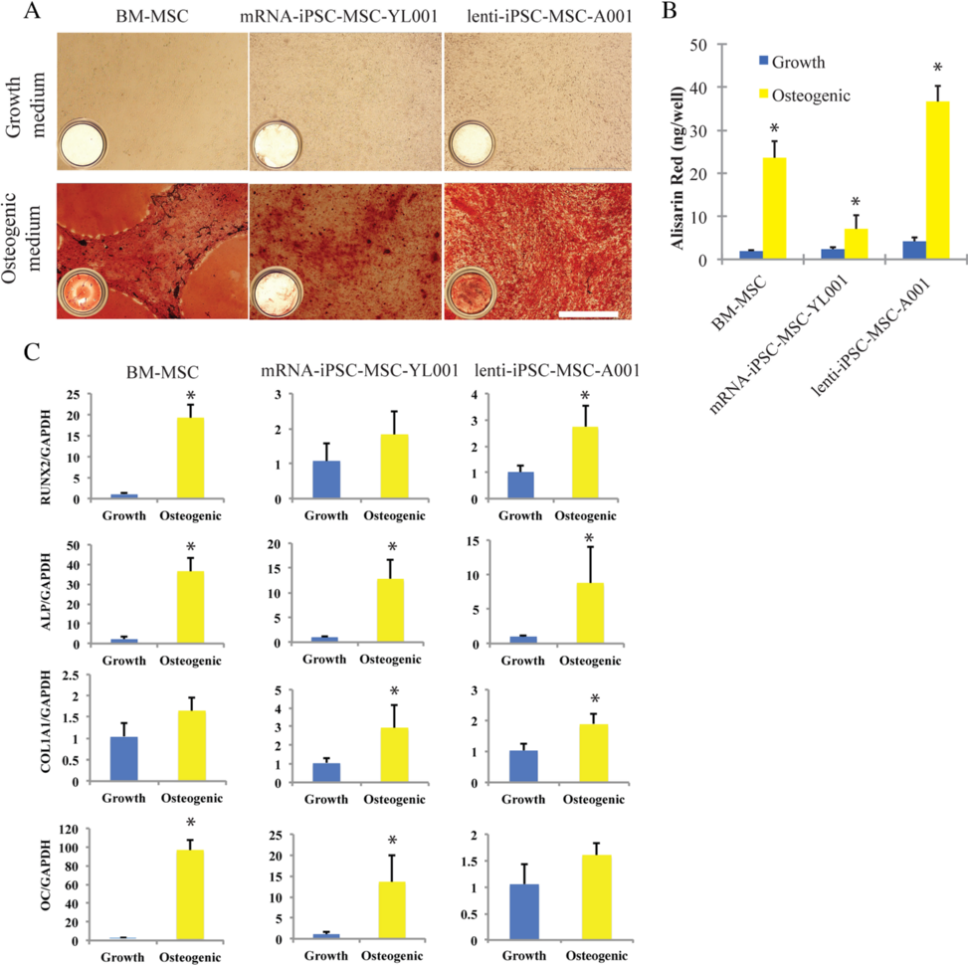
Comparative analyses of the osteogenic capacity of the iPSC-MSCs and BM-MSCs. **a** Alizarin Red staining of calcium in the BM-MSCs, the mRNA-iPSC-MSC-YL001, and the lenti-iPSC-MSC-A001 with growth medium or osteogenic medium for 3 weeks. Images are representative of six repeats. In the left corner there is an image from one well of the 96-well plate. Scale bar for the microscopic images is 300 μm. **b** Quantitative calcium deposition of the BM-MSC, mRNA-iPSC-MSC-YL001, and lenti-iPSC-MSC-A001 cells after 3 weeks in the MSC growth medium or osteogenic medium (n = 8). **c** Quantitative PCR analysis of the expression of osteogenic genes *RUNX2*, *ALP*, *COL1A1*, and *OC* in the BM-MSC, mRNA-iPSC-MSC-YL001, lenti-iPSC-MSC-A001 after 3 weeks in the MSC growth medium or osteogenic medium (n = 6). *P < 0.05. *BM* bone marrow, *iPSC* induced pluripotent stem cell, *MSC* mesenchymal stem cell

After 3 weeks of osteogenic induction, calcium mineralization was observed in all three MSC lines by Alizarin Red S staining (Fig. 4a). Mineralization was not observed in the MSCs cultured in normal growth medium. A more homogeneous and robust calcium staining was seen in the lenti-iPSC-MSC-A001 than in the mRNA-iPSC-MSC-YL001. This observation was further confirmed by quantification of calcium content (Fig. 4b). The lenti-iPSC-MSC-A001 line has an efficacy of osteogenesis similar to BM-MSCs. However, approximately twofold less osteogenic capacity was found in the mRNA-iPSC-MSC-YL001 cultures, which is likely due to less positivity of CD90 (Fig. 2) [25]. Increased expression of four osteogenic genes (runt-related transcription factor 2 (*RUNX2*), alkaline phosphatase (*ALP*), collagen type I alpha 1 (*COL1A1*), and osteocalcin (*OC*)) was detected in all MSCs after 3 weeks of osteogenic differentiation (Fig. 4c). The induced expression of *RUNX2*, *ALP* and *OC* was more robust in BM-MSCs relative to the iPSC-MSCs, whereas *Col1a1* was more strongly expressed in the iPSC-MSC cultures. Taken together, these results suggest that iPSC-MSCs derived with our method can achieve an adequate osteogenic efficacy similar to that of BM-MSCs.

**Fig. 5:**
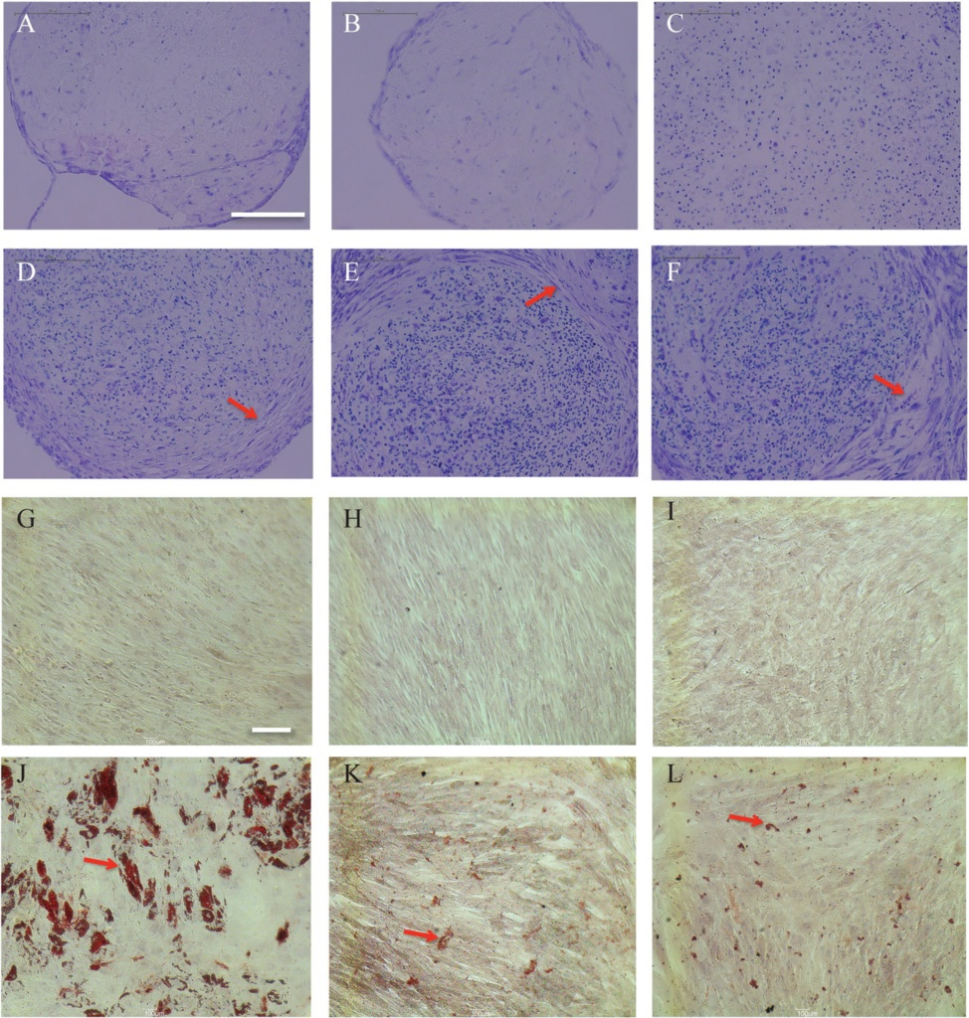
Comparative analysis of the chondrogenic and adipogenic capacities. Toluidine blue staining of cartilaginous extracellular matrix of the BM-MSCs (**a** and **d**), the mRNA-iPSC-MSC-YL001 (**b** and **e**), and the lenti-iPSC-MSC-A001 (**c** and **f**) after 3 weeks in MSC growth medium (**a**–**c**) or chondrogenic medium (**d**–**f**). Oil-Red O staining of lipids in the BM-MSCs (**g** and **j**), the mRNA-iPSC-MSC-YL001 (**h** and **k**), and the lenti-iPSC-MSC-A001 (**i** and **l**) after 3 weeks in MSC growth medium (**g**–**i**) or adipogenic medium (**j**–**l**). *Arrow heads* indicate the cartilaginous extracellular matrix (**d**–**f**) and lipids (**j**–**l**). Scale bar: **a**–**f**, 150 μm; **g**–**l**, 300 μm

For chondrogenic differentiation, the lenti-iPSC-MSC-A001, mRNA-iPSC-MSC-YL001 and BM-MSC lines were grown in a “V” shape 96-well plate according to a protocol described previously [26]. Toluidine blue staining of pellet sections was used to assess chondrogenic differentiation. Cartilaginous extracellular matrix is stained purple while undifferentiated or fibrous tissue is stained blue. After 3 weeks of chondrogenic differentiation, cartilaginous extracellular matrix was detected in all MSC lines cultured in chondrogenic medium (Fig. 5d–f), but not in growth medium (Fig. 5a–c). The lenti-iPSC-MSC-A001 and mRNA-iPSC-MSC-YL001 had formed similar cartilaginous structures compared with the BM-MSCs.

The adipogenic potential of the iPSC-MSCs was also characterized by in vitro adipogenic differentiation. Lipid droplets formed by adipogenesis were assessed by Oil Red O staining. After 3 weeks of adipogenic differentiation, lipid droplets were formed in both iPSC-MSC lines and the BM-MSCs in the adipogenic differentiation medium (Fig. 5j–l) but not in the growth medium (Fig. 5g–i). However, the adipogenic efficacy of both iPSC-MSC lines was much lower than the BM-MSCs. Lipid droplets from iPSC-MSCs were much smaller than those from BM-MSCs.

### The iPSC-MSCs and tri-lineage differentiated cells lack expression of stemness genes

**Fig. 6:**
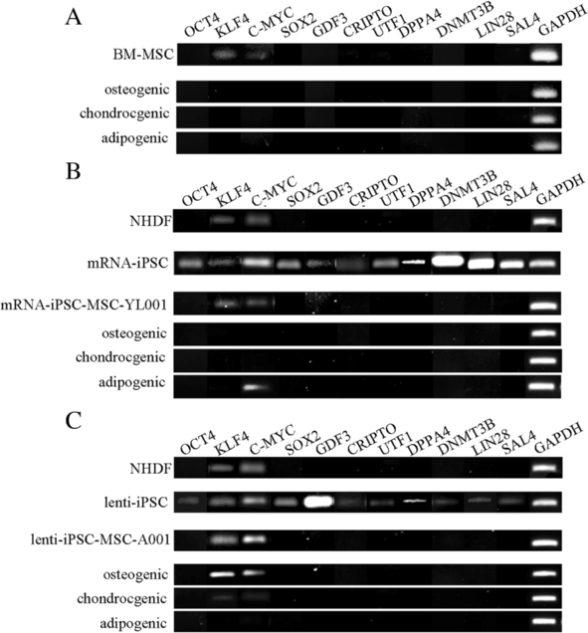
Gene expression analysis of “stemness” genes in the NHDFs, iPSCs (two lines: mRNA-iPSC and lenti-iPSC), BM-MSCs, mRNA-iPSC-MSC-YL001, lenti-iPSC-MSC-A001 and the three derived lineages (osteogenic, chondrogenic, adipogenic). Expression of eleven stemness genes (POU class 5 homeobox 1 (*OCT4*/ *POU5F1*), Kruppel-like factor 4 (*KLF4*), v-myc avian myelocytomatosis viral oncogene homolog (*C*-*MYC*), SRY (sex determining region Y)-box 2 (*SOX2*), growth differentiation factor 3 (*GDF3*), teratocarcinoma-derived growth factor 1 (*CRIPTO*/ *TDGF1*), undifferentiated embryonic cell transcription factor 1 (*UTF1*), developmental pluripotency associated 4 (*DPPA4*), DNA (cytosine-5-)-methyltransferase 3 beta (*DNMT3B*), lin-28 homolog A (*LIN28A*) and spalt-like transcription factor 4 (*SALL4*)) was analyzed by PCR. **a** BM-MSCs in growth medium, and after 3 weeks in osteogenic, chondrogenic and adipogenic medium; **b** NHDF, mRNA-iPSCs, mRNA-iPSC-MSC-YL001 in growth medium, and mRNA-iPSC-MSC-YL001 after 3 weeks in osteogenic, chondrogenic and adipogenic medium; **c** NHDF, lenti-iPSCs, lenti-iPSC-MSC-A001 in growth medium, and lviPSC-MSC-A001 after 3 weeks in osteogenic, chondrogenic and adipogenic medium. *BM* bone marrow, *iPSC* induced pluripotent stem cell, *MSC* mesenchymal stem cell, *NHDF* normal human dermal fibroblast

Loss of “stemness” in iPSC-MSCs and iPSC-MSC-derived lineages is one crucial safety criterion when applying iPSC-MSCs to tissue engineering and cell therapy. Lack of tumorigenic potential of the mRNA-iPSC-MSC-YL001 line was validated previously [16]. We further characterized the loss of “stemness” in the iPSC-MSC lines by analyzing the expression of 11 stemness genes (*OCT4*, *KLF4*, *C*-*MYC*, *SOX2*, *GDF3*, *CRIPTO*, *UTF1*, *DPPA4*, *DNMT3B*, LIN28A and *SALL4*) in the original iPSCs, iPSC-MSCs, and tri-lineage differentiated cells (osteogenesis, chondrogenesis, and adipogenesis). All stemness genes were expressed in the iPSCs, whereas most stemness genes, except *Klf4* and *C*-*Myc*, were not expressed in the iPSC-MSCs and the tri-lineage differentiated cells (Fig. 6). We also found that *Klf4* and *c*-*Myc* were expressed in the NHDF, which is similar to what has been reported previously [8, 27].

## Discussion

With technical improvements in generating pluripotent stem cells, differentiation of iPSCs to functionally specific cell types remains a major challenge. MSCs are a promising cell type for personalized therapies such as tissue engineering [28], immunosuppressive biotherapy [29], and regenerative organ repair [30]. We have previously developed a simple protocol to derive iPSC-MSCs and applied them for bone tissue engineering in synthetic three-dimensional scaffolds and nanofibers [4, 16]. Here, we compared two iPSC-MSC lines (derived with our protocol) to naïve BM-MSCs. This study demonstrated that the iPSC-MSCs derived by our protocol display many similar characteristics relative to the BM-MSCs, including fibroblast-like morphology, expression of typical mesenchymal markers, the capacity to efficiently differentiate into osteogenic and chondrogenic lineages, and loss of pluripotency. This study further supports our previous finding of efficient osteogenic potential for iPSC-MSCs in three-dimensional scaffolds and nanofibers [4, 16].

There are several advantages of using iPSC-MSCs for MSC-based therapies as compared to primary MSCs. The high proliferative capability iPSC-MSCs proven by this study (Fig. 3) and others [31–33], as well as the infinite self-renewal capacity of iPSCs [8], increase the potential of MSC-based cell therapies, which commonly requires repetitive administration of a large number of MSCs. Although MSCs can be easily isolated from various sources of adult tissues, such as BM, fat, placenta, blood, spleen, heart, pancreas, joints and so forth [34–36], this repetitive MSC isolation procedure would cause pain and risk of infection for patients. Furthermore, for certain patients with inherited genetic problems or age-related disorders, iPSC-MSCs would represent the best solution for gene- and cell-based therapy. One such example is Osteogenesis imperfect (OI), which is caused by dominant mutations of the type one collagen genes. With an adeno-associated virus-mediated gene targeting strategy, Deyle et al. generated iPSC-MSCs derived from OI patients and subsequently inactivated the mutated *COL1A1* and *COL1A2* genes in the iPSC-MSCs. The targeted iPSC-MSCs were able to restore normal collagen production and bone formation [37].

In terms of osteogenic and chondrogenic capacities of the iPSC-MSCs, we now have further confirmed their adequate efficacy relative to BM-MSCs [4, 16]. Although only the osteogenicity was thoroughly investigated in this study due to our research focus, the chondrogenicity of iPSC-MSCs has been systematically confirmed by studies from other research groups [38, 39].

However, this study also showed that both iPSC-MSC lines were less efficient with respect to adipogenic differentiation relative to their osteogenicity and chondrogenicity (Fig. 5). Although both iPSC-MSC lines derived by our protocol were capable of adipogenesis [16], the adipocytes and lipid droplets were much smaller in the cultures of iPSC-MSCs compared with those of BM-MSCs. Similarly, iPSC-MSCs derived by other methods have been reported to have less adipogenicity than BM-MSCs [32, 40]. A similar observation was reported for MSCs derived from human ESCs [41]. It is still unknown why MSCs derived from iPSCs or ESCs show less commitment towards adipogenicity. Many intrinsic factors could affect the adipogenicity of iPSC-MSCs, such as the iPSC-MSC derivation and cultivation methods, iPSC-MSC purity, and tissue of origin. Hynes et al. recently compared iPSC-MSCs originating from three different tissues: gingiva, periodontal ligament, and lung [42]. Although the efficacy of adipogenesis of those iPSC-MSCs was not assessed in their study, various capacities were observed among the three iPSC-MSCs lines, suggesting the notion that the tissue of origin plays a vital role in commitment differentiation. In our study, the lenti-iPSC-MSC-A001 had a higher adipogenic capacity than the mRNA-iPSC-MSC-YL001. However, both iPSC-MSCs lines were derived from dermal fibroblasts. Thus, the adipogenic difference may be due to clonal variation, which has been reported in other studies [42, 43].

According to ClinicalTrail.gov to date, there are 452 studies registered for “mesenchymal stem cells”, 32 studies registered for “induced pluripotent stem cells”, and 27 studies registered for both “mesenchymal stem cells” AND “induced pluripotent stem cells”. One key concern when using iPSCs and MSCs derived from iPSCs for cell therapy is their potential for teratoma formation. We have shown that the iPSC-MSCs generated by our method do not form teratoma after transplanting into immune deficient mice [16]. In this study, we further characterized a few “stemness” genes in the iPSC-MSCs. Except for *KLF4* and *C*-*MYC*, the rest of the “stemness” genes (*OCT4*, *SOX2*, *GDF3*, *CRIPTO*, *UTF1*, *DPPA4*, *DNMT3B*, *LIN28A*, *SALL4*) were not expressed in the iPSC-MSC lines, further implicating the safety aspect of the iPSC-MSCs. *KLF4* and *C*-*MYC* were also expressed in the BM-MSCs and in primary fibroblasts. Similarly, *KLF4* and *C*-*MYC* were reported to be expressed in human fibroblasts by a previous study [8], suggesting that the presence of *KLF4* and *C-MYC* should not contribute to teratoma formation by the iPSC-MSCs. With more studies conducted to characterize the safety and efficacy aspects of iPSC-MSCs, increasing numbers of clinical trials using iPSC-MSCs are expected in the future. We also observed an expression difference for *KLF4* and *C*-*MYC* between the mRNA-iPSC-MSC-YL001 and lenti-iPSC-MSC-A001 after osteogenic and adipogenic differentiation. Since *KLF4* and *C*-*MYC* are expressed in mRNA-iPSC-MSC-YL001 and lenti-iPSC-MSC-A001 cells, the expression of *KLF4* and *C*-*MYC* in osteogenic and adipogenic samples might have resulted from the proportion of parental iPSC-MSCs that failed to differentiate. The difference between the two iPSC-MSC lines could have resulted from the different mesenchymal heterogeneity of these two iPSC-MSCs as shown in Fig. 2.

Another important challenge for generating clinically compliant iPSC-MSCs is the time-effective generation of a large amount of functional MSCs. Several methods have been described to derive functional MSCs from human iPSCs. These methods vary from each other in terms of ingtermediate embryoid bodies, surface coating of tissue culture vessels, differentiation medium, and supplement of pathway inhibitors (e.g., the p38 MAPK inhibitor, SB203580 and the transforming growth factor-β pathway inhibitor SB431542) [32, 44]. The use of molecule SB431542 did not improve the adipogenic capacity of iPSC-MSCs [32]. However, robust adipogenesis of iPSC-MSCs has been achieved by supplementing the SB203580 inhibitor [44]. Our method for iPSC-MSC derivation involves neither intermediate embryoid bodies nor the use of pathway inhibitors. The derivation of iPSC-MSCs is achieved by MSC medium-mediated pre-differentiation, followed by MSC enrichment by serial passaging in MSC culture condition, which take approximately 1 month [4, 16]. A similar method has been applied to derive functional MSCs by other groups [42, 45]. Compared to the embryoid body-based differentiation method, this method is more time- and cost-effective, and compliant with large-scale production. However, as proved in this study, further improvements are still required for our iPSC-MSC method to generate functional MSCs that can resemble the naïve BM-MSC functions.

## Conclusions

The iPSC-MSCs derived by our method display most mesenchymal characteristics similar to adult BM-MSCs. The iPSC-MSCs achieve robust osteogenicity and chondrogenicity, but have very limited adipogenicity. Our results suggest that these iPSC-MSCs present a promising alternative cell source for MSC-based therapeutic applications.

## Abbreviations

BM: Bone marrow
BM-MSC: Bone marrow-derived mesenchymal stem cell
BSA: Bovine serum albumin
DMEM: Dulbecco’s modified Eagle’s medium
ESC-MSC: Embryonic stem cell-derived mesenchymal stem cell
FBS: fetal bovine serum
iPSC: Induced pluripotent stem cell
iPSC-MSC: Induced pluripotent stem cell-derived mesenchymal stem cell
KSR: Knockout serum replacement
MSC: Mesenchymal stem cell
NHDF: Normal human dermal fibroblast
NPD: Number of population doublings
OI: Osteogenesis imperfect
P: Passage
PBS: phosphate-buffered saline
